# Performance of the Neonatal Tetanus Surveillance System (NTSS) in Sana'a, Yemen: Evaluation Study

**DOI:** 10.2196/27606

**Published:** 2021-05-04

**Authors:** Khaled Mohammed Al-Jamrah, Basheer Abdulgalil Al Nabehi, Khaled Abdullah Almoayed, Labiba Saeed Anam, Yousef S Khader

**Affiliations:** 1 Yemen Field Epidemiology Training Program Ministry of Public Health and Population Sana'a Yemen; 2 Neonatal Tetanus Surveillance Program Ministry of Public Health and Population Sana'a Yemen; 3 Department of Public Health, Community Medicine, and Family Medicine Faculty of Medicine Jordan University of Science & Technology Irbid Jordan

**Keywords:** neonatal tetanus, evaluation, surveillance, CDC guidelines, Yemen

## Abstract

**Background:**

The Neonatal Tetanus Surveillance System (NTSS) in Yemen was established in 2009 to identify high-risk areas, determine trends, and evaluate elimination activities. Since its launch, the NTSS had never been evaluated.

**Objective:**

This study aimed to assess the performance of NTSS and determine its strengths and weaknesses to recommend improvements.

**Methods:**

The US Centers for Disease Control and Prevention (CDC) guidelines were used for evaluating the NTSS. Stakeholders at the central, district, and facility levels were interviewed to rate the attributes of the NTSS. The percentage scores for attributes were ranked as poor (<60%), average (≥60% to <80%) and good (≥80%).

**Results:**

The overall usefulness score percentage was 38%, which indicates a poor performance. The performance of the NTSS was rated as average on flexibility (score percent: 68%) and acceptability (score percent: 64%) attributes and poor on stability (score percentage: 33%), simplicity (score percentage: 57%), and representativeness (score percentage: 39%) attributes. About 65% of investigation forms were completed within 48 hours of notification date. Data quality was poor, as 41% of the core variables were missing.

**Conclusions:**

The overall performance of the NTSS was poor. Most of the system attributes require improvement, including stability, simplicity, quality of data, and completeness of investigation. To improve the performance of NTSS, the following are recommended: capacity building of staff (focal points), strengthening NTSS through technical support and government funding to ensure its sustainability, establishing electronic investigation forms for improving the system data quality, and expansion of NTSS coverage to include all private health care facilities.

## Introduction

### Background

Neonatal tetanus (NT) is a life-threatening vaccine-preventable disease and one of the most underreported diseases in many developing countries. It is caused by the toxin of *Clostridium tetani*, a ubiquitous, spore-forming, gram-positive bacillus found in high concentrations in soil and animal excrement. The disease usually occurs in rural settings with poor access to health facilities, characterized by generalized rigidity and convulsive spasms of skeletal muscles that usually involve the jaw and neck and then become generalized [1]. Newborns can become infected through contaminated instruments used to cut the umbilical cord or by improper handling of the umbilical stump [1,2].

NT remains a major cause of infant and neonatal mortality in many developing countries, but it is preventable by immunization and/or assuring clean delivery and postdelivery practices [2]. According to the World Health Organization (WHO), an NT case is defined as “a neonate with the normal ability to suck and cry during the first 2 days of life, between 3 to 28 days of age cannot suck normally and becomes stiff or has spasms” [3]. Global elimination is defined as an annual rate of <1 case of NT per 1000 live births at the district level [4].

 Globally, good progress has been made in the last two decades. The number of reported NT cases worldwide declined by 90%, from 17,935 in 2000 to 1803 in 2018, and estimated NT deaths decreased by 85%, from 170,829 in 2000 to 25,000 in 2018 [4]. This disease accounts for 1% of worldwide neonatal mortality, compared with 7% in 2000 [5].

Despite the appreciable drop in the number of NT cases attributed to the global NT elimination strategies, NT still remains among the leading causes of death in 14 developing countries that have not yet eliminated NT [6]. The Eastern Mediterranean Region (EMR) has been ranked at the fourth position among WHO regions in term of NT burden. In 2018, 181 (10%) of NT cases were reported from 6 countries in the region: Afghanistan, Saudi Arabia, Iraq, Morocco, Egypt, and Yemen [4].

### Neonatal Tetanus Surveillance in Yemen

Yemen is one of the 5 remaining countries in the EMR that have not achieved the global elimination target set by WHO [6]. NT remains a public health problem in Yemen, where it is one of the major causes of neonatal mortality. In 2018, more than 64% of all NT cases in EMR were reported from Yemen. A total of 2069 NT cases were reported from 1980 to 2018 [4].

Tetanus immunization has been included in the national expanded program of immunization in Yemen since 1977, and includes 5 doses of tetanus toxoid vaccine for women of child-bearing age. Despite the availability of an inexpensive and effective tetanus vaccine, Yemen is far behind in tetanus toxoid vaccine coverage. Over the last decade, 17% to 21% of women received at least 2 doses of the tetanus toxoid vaccine in Yemen [7].

The Neonatal Tetanus Surveillance System (NTSS) was established in Yemen in 2009, with the objectives to identify high-risk areas, determine trends of NT, and evaluate tetanus elimination activities. NTSS in Yemen is a passive surveillance system that relies on the identification of NT cases collected at the health facility level and then reported to the next higher level at a specified frequency (weekly), even if there are zero cases (referred to as zero reporting). In addition, all NT cases are reported through community-based surveillance (CBS) and the Electronic Disease Early Warning System (eIDEWS). Particularly in high-risk areas, the NTSS relies on CBS through traditional birth attendants, community leaders, traditional healers, or other community members who are sensitized to report NT cases and deaths to health authorities. The eIDEWS network of reporting sites includes both public and private health facilities.

### Objectives

High-quality NT surveillance is a key component of the NT elimination strategy, and its data are used to identify areas or subpopulations at high-risk for NT and guide effective public health response for NT elimination. However, since its launch in Yemen, NTSS had never been evaluated. Therefore, this study aimed to assess the level of usefulness and performance of the NTSS attributes, determine its strengths and weaknesses, and suggest recommendations for improvement.

## Methods

### Study Design

A descriptive evaluation study was conducted to describe NTSS and its performance according to the Centers for Disease Control and Prevention (CDC) guideline for evaluating disease surveillance systems [8]. The study was performed in the city of Sana’a from October to December 2018.

### Study Population and Sampling

The study population involved 31 stakeholders: 5 from the central level, 3 from governorate level, 3 from districts level, and 20 health facility focal points. At the district and health facility levels, samples were selected using a multistage sampling method. Using simple random sampling, 30% (3/10) of the districts—Assabain, Ma’ain, and Assafi’yah—were selected. A total of 33% (20/60) of health facilities were selected by stratified random sampling distributed proportionally among the chosen districts according to the curative services (public and private) in each district. The selection of health facilities from each chosen district was based on simple random sampling.

### Data Collection

Desk review of the main NTSS documents and literature was conducted to describe the system. In depth interviews were conducted with NTSS stakeholders at the central and governorate levels to measure usefulness, flexibility, and stability. A semistructured questionnaire was used to collect data from surveillance coordinators of governorates, districts, and focal points at health facility levels to evaluate simplicity and acceptability (Multimedia Appendix 1). In addition, data registries from 2009 to 2018 were obtained to measure data quality, timeliness of investigation, completeness of investigation, and representativeness.

### Performance of the System

The indicators of the level of usefulness, flexibility, and stability attributes were assessed using yes or no (yes = 1; no = 0) questions. The indicators of simplicity and acceptability were assessed using a 5-point Likert scale (1 = strongly disagree, 2 = disagree, 3 = neutral, 4= agree, and 5 = strongly agree). Quality of data was assessed by measuring the percentages of missing variables. Timeliness of investigation was measured by the percentage of all suspected cases investigated within 48 hours of notification. Completeness of investigation was measured by the proportion of NT suspected cases reported that have been investigated. Representativeness was measured by the percentage of all public and private health facilities in Sana’a that were covered by NTSS.

### Data Analysis and Interpretation

The usefulness, flexibility, and stability indicators were scored as 0 or 1, whereas the simplicity and acceptability indicators were scored from 1 to 5. For each indicator, the score percentage was calculated as the following:

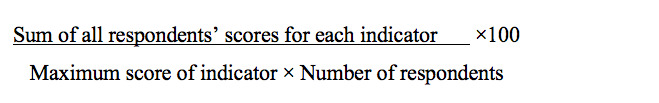



The overall attribute score percentage was calculated as the following:

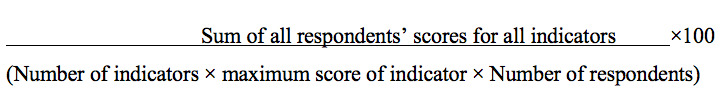



Each indicator and overall attribute score percentage was represented as score rank and interpreted as poor <60%, average ≥60% to <80%, or good ≥80%. Missing data were measured by selecting 1-year data to calculate the percentage of the missed core variables. The core variables included case identification, date of birth, sex, place of usual residence, date of illness onset, date of notification, date of investigation, symptoms in case definition, outcome (alive/dead), maternal vaccination history, place/type of delivery, tool used for cutting cord, and material applied to cord. For any case, if information on any of the core variables is missing, the investigation was considered inadequate.

Timeliness of investigation was calculated by dividing the number of suspected NT cases investigated within 48 hours of notification by the number of suspected NT cases investigated × 100. Completeness of investigation was calculated by dividing the number of NT case investigations by the number of suspected NT cases reported × 100. Representativeness was calculated by dividing the number of public and private health facilities in Sana’a that are covered by NTSS by the total number of public and private health facilities in Sana’a × 100. Epi Info (version 7.2, Division of Health Informatics and Surveillance, CDC) was used for data entry and analysis. Data were described using frequencies, score percentages, and score rank.

## Results

### Participant Characteristics

A total of 31 participants (61% [19/31] male and 39% [12/31] female) were interviewed from 4 levels: central, governorate, district, and health facility. The median of their ages was 42 years (range 24 to 65 years) and the median years of their experience in surveillance systems was 4.0 years (range 1 to 20 years).

### Level of Usefulness

Based on 8 in-depth interviews with stakeholders at central and governorate levels, the overall usefulness score percentage was 38%, which indicates a poor performance. Only one usefulness indicator (“The system data are used to estimate NT magnitude, incidence, and mortality rates”) was ranked as good (Table 1). One of the respondents said “Unfortunately, NT is neglected by researchers and the Ministry of Public Health and Population.”

**Table 1:** The score, score percentage, and rank of usefulness indicators of the Neonatal Tetanus Surveillance System in Sana’a, 2018 (n=8).

Indicator	Score	Score percentage	Rank
The system data are used to estimate NT^a^ magnitude, incidence, and mortality rates.	7	88	Good
The system data are used to monitor the trend of NT spread over time.	4	50	Poor
The system data are used to identify high-risk areas.	4	50	Poor
The system data are used to update and develop the national policy strategy for NT elimination.	1	13	Poor
The system data are used to assess the effect of interventions.	1	13	Poor
The system provides a basis for epidemiologic research.	1	13	Poor
Overall usefulness	18	38	Poor

^a^NT: neonatal tetanus.

### Flexibility

The overall score of flexibility was 68%, which reveals an average performance (Table 2). Three of the flexibility indicators (“The system can be adapted to accommodate new additional information” [eg, change in case definition], “The system can be adapted to increases in the reporting sources,” and “The system can be adapted to integrate with other surveillance”) had good rank. One indicator (“The system is not affected by fund variation”) was ranked as poor.

**Table 2:** The score, score percentage, and rank of flexibility indicators of the Neonatal Tetanus Surveillance System in Sana’a, 2018 (n=8).

Indicator	Score	Score percentage	Rank
The system can be adapted to accommodate new health-related events with little resources and time.	5	60	Average
The system can be adapted to accommodate new additional information (eg, change in case definition).	7	88	Good
The system is not affected by fund variation.	0	0	Poor
The system can be adapted to increases in the reporting sources.	7	88	Good
The system can be adapted to integrate with other surveillance.	8	100	Good
Overall flexibility	27	68	Average

### Stability

The overall stability of the system scored 33%, which reveals a poor performance. Of 5 stability indicators, only one (“The system does not require time for collecting, sending, receiving, and managing data”) had a good rank (Table 3). One of the respondents said “There is no qualified coordinator capable of working continuously, and the most important obstacle is the lack of funding.”

**Table 3:** The score, score percentage, and rank of stability indicators of the Neonatal Tetanus Surveillance System in Sana’a, 2018 (n=8).

Indicator	Score	Score percentage	Rank
The system has funds from nongovernmental organizations.	0	0	Poor
The system has governmental funds.	1	13	Poor
The system is stable without sponsor funds.	0	0	Poor
The system does not require time for collecting, sending, receiving, and managing data.	8	100	Good
The reports are released regularly.	4	50	Poor
Overall stability	13	33	Poor

### Simplicity

A total of 26 stakeholders at governorate, district, and health facility levels rated the simplicity. Of 9 indicators, 5 had poor ranking. Only one indicator had good ranking. The overall simplicity of the system scored 57%, which indicates a poor performance (Table 4).

**Table 4:** The score, score percentage, and rank of simplicity indicators of the Neonatal Tetanus Surveillance System in Sana’a, 2018 (n=26).

Indicator	Score	Score percentage	Rank
Case definition is available.	71	55	Poor
Case definition is easy to apply.	95	73	Average
Investigation forms are available.	48	37	Poor
Investigation forms are easy to complete.	52	40	Poor
Data collection does not require telephone contact or home visit.	84	65	Average
Data collection does not need much time.	99	76	Average
Data transportation to the central level is very easy.	108	83	Good
Received training on NT^a^.	35	27	Poor
Data updating and follow-up of cases are easy.	77	59	Poor
Overall simplicity	669	57	Poor

^a^NT: neonatal tetanus.

### Acceptability

The acceptability indicator related to willingness to participate in NTSS had good rank. However, satisfaction with NTSS was poor. The overall acceptability score percentage was 64%, which indicates an average performance.

### Timeliness and Completeness of Investigation

Of reported cases, the overall completeness of routine reporting under passive surveillance was only 23% (120/524), which indicates a poor performance. A total of 65% (340/524) of investigation forms were completed within 48 hours of the notification date, indicating an average timeliness performance, while 10% (53/524) of investigation forms were completed more than 48 hours after the notification date. The remaining (131/524, 25%) had no investigation date.

### Data Quality

The data quality was poor, with 41% (645/1572) of the core variables missing. The highest percentage of data missing was in maternal vaccination history (96/131, 73%), followed by material applied to cord (72/131, 55%). The lowest percentage of missing was in date of birth (14/131, 11%) followed by sex variable (6/131, 5%).

### Representativeness

Almost all (64/66, 97%) public health facilities and 26.7% (80/300) of private health facilities were covered by NTSS. The overall representativeness score percentage was only 39%, that indicates a poor representativeness.

### Strengths and Weaknesses of the NTSS

The strengths of the system include easy data collection; transfer through eIDEWS; existence of surveillance staff in governorate, district, and health facility levels; several sources of reporting such as eIDEWS and CBS; and existence of new qualified leadership.

The weaknesses of the system include lack of investigation forms in the health facilities; no activities for the NTSS; lack of central supervision and communication; and no brochures, posters, or publications available on NT.

## Discussion

### Principal Findings

Every disease surveillance system should be analyzed and evaluated periodically to ensure it meet its objectives and improve performance wherever necessary. This evaluation revealed contradictory views about the usefulness of the NTSS, where it was generally considered poor. For example, the surveillance system did not play any role in assessing the effectiveness of the NT elimination strategy and was not capable of identifying areas at high risk. However, the surveillance data were useful to estimate NT magnitude, incidence, and mortality rates, which are the main goals for NTSS. The system might be considered useful if it satisfactorily addresses at least one of the usefulness indicators [8].

The NTSS demonstrated average flexibility. This result is reasonable, particularly with indicators related to the change in case definition, reporting sources, and integration with other surveillance. Receiving data from several sources such as eIDEWS and CBS may enhance the flexibility of the system. However, flexibility may be negatively affected by fund variation. Similar findings were reported by another study, which revealed its flexibility by incorporating information on measles and acute flaccid paralysis systems [9]. In the other hand, our finding disagrees with those of a previous evaluation performed in Baluchistan, Pakistan, which demonstrated that the system had poor flexibility [10].

Despite the ability of the system to collect, send, receive, and manage data with little resources and time, the stability of NTSS was poor. This poor stability of NTSS reflected the weak capability of the system to release reports regularly and lack of funds, especially governmental funds, which affect the system activities implementation. Therefore, the system is unstable and its sustainability is in danger.

Concerning the simplicity, the study results showed that NTSS simplicity was generally poor. This result might be due to the unavailability of case definition and investigation forms, as well as poor training, which can be attributed to lack of funds necessary for implementation of system activities. However, the transfer of the data to the central level was simple.

The findings showed that the acceptability of NTSS was average, as the majority of respondents were willing to participate in the surveillance system. Nevertheless, about two-thirds of respondents were unsatisfied with NTSS. This might be due to lack of interaction between the central and health facility levels, and this may result in lack of communication. Similarly, previous evaluation performed in Baluchistan, Pakistan, found that the system was acceptable for all stockholders with average rank [10].

Completeness of reporting for NT depends on conscientious notification, use of health services, health-seeking behavior, access to health services, [11] and a sensitive surveillance system [3,12].

Our findings revealed the completeness of investigation forms was poor. This result was far from the WHO target for completeness of investigation (≥90%) [3]. It might be due to unavailability of investigation forms, registers, and case definitions in the health facilities as well as lack of central supervision, communication, and training courses for staff. A similar study suggested that the availability and widespread use of birth attendant logs, vital events registries, and standard case definitions in reporting sites may impact NT reporting and surveillance [13].

The NTSS has average timeliness of investigation within 48 hours after notification. This result was lower than the WHO target for timeliness of investigation, which is ≥80% [3]. This finding might be due to the investigation date being missing in a quarter of the investigation forms.

High-quality surveillance data and other key program indicators should be used to monitor the impact of interventions, achievement, and maintenance of NT elimination [3]. The findings of this evaluation showed poor quality of NTSS investigation-completed forms. In addition, the percentage of missing data was high, especially data related to the maternal vaccination history and material applied to cord. In contrast, the percentage of missing data was low on the date of birth and sex variables. This might be due to lack of staff training and incentives. In comparison, a previous evaluation in Baluchistan, Pakistan, also demonstrated that the system had poor data quality [10].

The representativeness of NTSS in general was poor in Sana’a, particularly among private health facilities. However, the representativeness of NTSS was good among public health facilities.

### Limitations

This evaluation has some limitations. This evaluation was limited to Sana’a due to time, budget, and security constraints. Moreover, arrival time of the investigation forms was not measured because it was not included in the investigation forms.

### Conclusion

The overall performance of the NTSS was poor, and it is not meeting its objectives. Most of the system attributes require improvement, such as usefulness, stability, simplicity, quality of data, and completeness of investigation. In contrast, the system flexibility, acceptability, and timeliness of investigation were average. The representativeness of NTSS was poor in Sana’a, particularly among private health facilities. The most important reasons for system weakness were lack of governmental funds, program activities, staff training, central communication, and supervision. To improve the performance of NTSS, the following are recommended: capacity building of staff (focal points) by regular trainings, frequent quality checks through field visits, strengthening NTSS through technical support and government funding to ensure its sustainability, establishing electronic investigation forms for improving the system data quality, and expansion of NTSS coverage to include all private health care facilities.

## Appendix

Multimedia Appendix 1 Data collection questionnaire.

## Abbreviations

CBS: community-based surveillance
CDC: Centers for Disease Control and Prevention
eIDEWS: Electronic Disease Early Warning System
EMR: Eastern Mediterranean Region
NT: neonatal tetanus
NTSS: Neonatal Tetanus Surveillance System
WHO: World Health Organization
