# Supplementary material for: Performance of the Neonatal Tetanus Surveillance System (NTSS) in Sana'a, Yemen: Evaluation Study
Source: JMIR Public Health Surveill. 2021 May 4;7(5):e27606. doi: 10.2196/27606 (PMC8132981; doi:10.2196/27606)
Supplement: Multimedia Appendix 1 [file publichealth_v7i5e27606_app1.docx]

**Appendix 1. Performance of the Neonatal Tetanus Surveillance System (NTSS) in Sana'a, Yemen: Evaluation Study**

**A: (Central and governorate levels, questionnaire)**

**No:** _____________ **Age:** ________  **Sex: __________**

**Job:** ____________ **Experience:** ________ **Date**: ________

**1. Usefulness**

Does the NTSS data used to estimate NT magnitude, incidence and mortality rates?

0-No 1-Yes

**Comment:**

Does the data used to monitor the trend of NT spread over time?

0-No 1-Yes

Does the data used to identify areas at high risk?

0-No 1-Yes

Does the data used to update & develop the national policy strategy for NT elimination?

0-No 1-Yes

**Comment:**

Does the data used to assess the effect of interventions?

0-No 1-Yes

**Comment:**

Does the system provide a basis for epidemiologic research?

0-No 1-Yes

**Comment:**

- 1. **Flexibility**

Does the system adapted to accommodate to new health related events with little resources and time?

0-No 1-Yes

**Comment:**

Does the system adapted to accommodate to new additional information (e.g. change in case definition)?

0-No 1-Yes

**Comment:**

Does the System not affect by fund variation?

0-No 1-Yes

**Comment:**

Does the system adapted to increase in the reporting sources?

0-No 1-Yes

**Comment:**

Does the system adopt to integrate with other surveillance?

0-No 1-Yes

**Comment:**

**3. Stability**

Does the system has funds from non-governmental organization?

0-No 1-Yes

Does the system have governmental fund?

0-No 1-Yes

Do you think the system is stable without sponsors fund?

0-No 1-Yes

Do you think the system not required time to collecting, sending, receiving and manage data?

0-No 1-Yes

Are the reports released regularly?

0-No 1-Yes

What are the strengths you have in the system?

What are the weaknesses you have in the system?

Do you have any suggestions or recommendations about the program?

**Thank you**

**B: (Governorate, Districts and health facilities levels, questionnaire)**

**No:** _____________ **Age:** ________  **Sex: __________**

**Job:** ____________ **Experience:** ________ **District**: ________

|  |  |  | **1** | **2** | **3** | **4** | | **5** | **Comment** |
| --- | --- | --- | --- | --- | --- | --- | --- | --- | --- |
|  |  |  | **Strongly disagree** | **Disagree** | **Neutral** | **Agree** | | **Strongly Agree** |  |
| **Simplicity** |  | Case definition is available |  |  |  |  | |  |  |
|  |  | Case definition is easy to apply |  |  |  |  | |  |  |
|  |  | Investigation forms always available |  |  |  |  | |  |  |
|  |  | Investigation format is easy to fill |  |  |  |  | |  |  |
|  |  | Collecting detailed information of reported cases don’t require telephone contact or home visit |  |  |  |  | |  |  |
|  |  | Collecting data do not need much time |  |  |  |  | |  |  |
|  |  | Data transportation to the central level is very easy |  |  |  |  | |  |  |
|  |  | You received training on NT |  |  |  |  | |  |  |
|  |  | The updating of data and follow up of cases are easy |  |  |  |  | |  |  |
|  | **Total Score =** | |  |  |  |  | |  |  |
| **Acceptability** |  | You are willing to participate within the NTSS |  |  |  | |  |  |  |
|  |  | You are completely satisfied with NTSS |  |  |  | |  |  |  |
|  | **Total Scores =** | |  |  |  | |  |  |  |

1. **What are the strengths you have in the system?**
2. **What are the weaknesses?**
3. **Any suggestion or recommendations you want to add.**

**Thank you**
